# Experimental Investigation of the Effect of Implanting TiO_2_-NPs on PVC for Long-Term UF Membrane Performance to Treat Refinery Wastewater

**DOI:** 10.3390/membranes10040077

**Published:** 2020-04-21

**Authors:** Faris H. Al-Ani, Qusay F. Alsalhy, Rawia Subhi Raheem, Khalid T. Rashid, Alberto Figoli

**Affiliations:** 1 Civil Engineering Department, University of Technology, Alsinaa Street 52, Baghdad 10066, Iraq; farishamodi@gmail.com (F.H.A.-A.); rawiasubhi826@gmail.com (R.S.R.); 2 Membrane Technology Research Unit, Chemical Engineering Department, University of Technology, Alsinaa Street 52, Baghdad 10066, Iraq; 80007@uotechnology.edu.iq; 3 Institute on Membrane Technology, National Research Council (ITM-CNR), 87030 Rende (CS), Italy; a.figoli@itm.cnr.it

**Keywords:** composite membranes, ultrafiltration application, TiO_2_NPs, PVC, oily wastewater, crossflow filtration

## Abstract

This study investigated the impact of implanting TiO_2_-NPs within a membrane to minimize the influence of long-term operation on the membrane characteristics. Four poly vinyle chloride-titanium oxide (PVC-TiO_2_-NPs) membranes were prepared to create an ultrafiltration membrane (UF) that would effectively treat actual refinery wastewater. The hypothesis of this work was that TiO_2_-NPs would function as a hydrophilic modification of the PVC membrane and excellent self-cleaning material, which in turn would greatly extend the membrane’s lifetime. The membranes were characterized via Fourier transforms infrared spectroscopy (FTIR), X-ray diffraction (XRD), energy dispersive X-ray (EDX), atomic force microscope (AFM), and scanning electron microscope (SEM). The removal efficiency of turbidity, total suspended solid (TSS), oil and grease, heavy metals and chemical oxygen demand (COD) were investigated. Contact angle (CA) reduced by 12.7% and 27.5% on the top and bottom surfaces, respectively. The PVC membrane with TiO_2_-NPs had larger mean pore size on its surface and more holes with larger size inside the membrane structure. The addition of TiO_2_-NPs could remarkably enhance the antifouling property of the PVC membrane. The pure water permeability (PWP) of the membrane was enhanced by 95.3% with an increase of TiO_2_ to 1.5 gm/100gm. The PWP after backwashing was reduced from 22.3% for PVC to 10.1% with 1.5 gm TiO_2_-NPs. The long-term performance was improved from five days for PVC to 23 d with an increase in TiO_2_-NPs to 1.5 gm. The improvements of PVC-TiO_2_-NPs long-term were related to the enhancement of the hydrophilic character of the membrane and increase tensile strength due to the reinforcement effect of TiO_2_-NPs. These results clearly identify the impact of the TiO_2_-NPs content on the long-term PVC/TiO_2_-NPs performance and confirm our hypothesis that it is possible to use TiO_2_-NPs to effectively enhance the lifetime of membranes during their long-term operation.

## 1. Introduction

In recent years, drinking water scarcity has become a global crisis, in large part as a result of inadequate conventional treatment techniques. Not only were these techniques not efficient, but they resulted in toxic substance by-products, causing several million people to lose their lives from various diseases annually [1] Wastewater is a by-product of domestic, industrial, commercial, or agricultural activities. Types of wastewater include domestic wastewater from households, municipal wastewater from communities (also called sewage), and industrial wastewater [2]. Rapid industrial growth, such as in metallurgical, pharmaceutical, petrochemical, oil and gas, and food industries, has led to a large production of oily wastewater. 

Unfavorable compounds produced during oil production and refining negatively affect the environment. Oily wastewater is one of the largest sources of unfavorable compounds. Oil extraction and refining operations are accompanied by large amounts of contaminants [3]. 

There are several techniques for the purification of the wastewater produced by the petroleum industry, including conventional physical, chemical, and biological methods, although each has its own drawbacks. As result of these disadvantages, researchers are investigating the use of membrane-based technologies for wastewater treatment applications. However, the limitations of conventional polymeric membranes have led to the addition of inorganic fillers to enhance membrane performance and to overcome fouling phenomena. The latest development is to enhance membranes with nanoparticles for use in water treatment [4]. 

Among the many polymer types used for the preparation of membranes, PVC is a highly versatile polymer compatible with many additives. It is classified as a chemically stable material that is resistant to acid, alkali, and almost all inorganic chemicals, and it also produces a tiny improvement in the mechanical strength [5]. Among different nanoparticles used as additives, such as carbon nanotubes [3,6], zinc oxide (ZnO-NPs) [4,7], graphene oxide [3] and titanium dioxide [8], TiO_2_ has received the most attention due to its stability under harsh conditions, its commercial availability, and its ease of preparation. The addition of nanoparticles into the casting solution has provided various advantages, e.g., improving the hydrophilic character, minimizing the fouling phenomena, and improving the thermal stability and mechanical properties of the composite membranes. Several scientists have attempted to minimize the fouling phenomena of polymeric membranes by adding different amounts of titanium oxide nanoparticles (TiO_2_-NPs) into various polymer matrices, for example, polysulfone (PSF), polyethersulfone (PES), polypropylene (PP), polyvinylidene fluoride (PVDF), and cellulose acetate (CAC) [9]. Yu et al. [10] added silver-embedded, nanosized titanium dioxide (Ag-n-TiO_2_) particles to PVC membranes to improve the anti-biofouling properties of these membranes by using synthetic bacterial solutions. It was found that the hydrophilicity, permeability, and retention capability increased with Ag-n-TiO_2_ concentrations, with an enhancement in their resistance to biofilm formation. Abedini et al. [11] prepared TiO_2_-CAC membranes with various contents of TiO_2_. The pure water permeability (PWP) was enhanced by adding nanoparticles. Jamed et al. [6] studied the influences of embedding functionalized multi-walled carbon nanotubes (MWCNTs) and alumina on the performance of membrane distillation for synthetic saline water desalination. The embedding of functionalized MWCNTs led to a positive modification of the membrane properties. Alsalhy et al. [7] investigated the impact of ZnO-NPs on polyphenylsulfone (PPSU) membranes properties, which is used for treatment of an aqueous solution of dye (Fw Direct red 80; Mw = 1373.09). The membrane hydrophilicity and performance were improved with the addition of ZnO-NPs, with no important change in the rejection of dyes. Alsalhy et al. [5] prepared anti-biofouling PVC-ZnO-NPs membranes to apply to actual hospital wastewater treatment using a submerged membrane bioreactor (SMBR). The addition of ZnO-NPs led to a great increase in the roughness and the hydrophilicity of the membrane. Both the lifetime of the membrane and its PWP improved greatly. Sadiq et al. [3] investigated using a PVC-MWCNT-g-GO ultrafiltration membrane to remove chemical oxygen demand (COD) from actual wastewater from the Al-Dura refinery (Bagdad, Iraq). The membrane exhibited increases in water flux as the MWCNT-g-GO content increased. Yuliwati et al. [12] prepared PVDF ultrafiltration membranes using hydrophilic and pore former additives for refinery produced wastewater treatment. Titanium dioxide (TiO_2_) was used as a hydrophilic additive. The membrane hydrophilicity was enhanced with the addition of a low concentration of TiO_2_ nanoparticles. The membrane exhibited 82.5 L/m^2^·h maximum flux and 98.83% rejection of refinery wastewater. Various studies reporting the influences of NPs on the different polymers are summarized in Table 1.

Recently, Behboudi et al. [20] prepared a PVC-TiO_2_ ultrafiltration membrane (UF) membrane and found that the hydrophilic character and the antifouling properties of the PVC-TiO_2_ membranes by filtration of synthetic Bovine serum albumin (BSA) solution and the PWP were greatly enhanced. Mcgaughey et al. [21] found significant variations in the morphology of the membrane surface that reduced the hydrophobic character of both the external and internal membrane during the membrane distillation (MD) operation of synthetic moderate to high salinity solutions after 100 days. They also observed that the contact angles decreased by 56% and 26% on the permeate and feed faces, respectively. After 20 days of MD operation, the morphological character changed in a negative way. 

Due to long-term reuse, the membrane exposure to cleaning agents at various operating conditions negatively impacts the characteristics of the membrane; thus, the performance of the membranes may suffer. Therefore, the study of membrane lifetimes is essential to ensure that the membrane does not deteriorate during long-term operation [5].

Therefore, in spite of the frequent use of TiO_2_-NPs as an enhancer to membrane performance, the impact of implanting TiO_2_-NPs within the membrane body to minimize the influence of long-term operation on the performance of the composite membrane has not yet been studied extensively. The practicality importance of the present work was to ensure that the membrane does not deteriorate during the long-term operation by implanting TiO_2_-NPs in the membranes wall. The hypothesis of this study was to use the TiO_2_-NPs as anti-fouling particles to overcome the possible changes in characteristics of the PVC-TiO_2_-NPs membrane that occur during operation, which in turn greatly extend the membrane long-term. The results of the present work were used to better understand the effect of long-term operation on the performance of the membranes. The major aim of this research is to evaluate the effect of TiO_2_-NPs content in the casting solution on changes in the performance efficiency of the membrane after long-term operation. To clearly identify the impact of the TiO_2_-NPs content on the long-term UF membrane performance, actual oily wastewater from the Al-Dura refinery was used. Three amounts of TiO_2_-NPs were added to the casting solution: 0.5, 1, and 1.5 gm. The PVC-TiO_2_-NPs membranes were characterized using SEM, AFM, FTIR, EDX, contact angle, tensile strength, thickness, and porosity. The efficiency of the PVC-TiO_2_-NPs UF membranes was investigated by measuring the permeation flux and the membrane’s ability to remove turbidity, total suspended solid (TSS), oil and grease, heavy metals, and COD. After use, the membranes with implanted nanoparticles were analyzed with UF experiments to understand the nanoparticles’ influence on the composite membrane performance during long-term operation. 

## 2. Experimental Work

### 2.1. Materials

PVC resin (65 kg/mol) was purchased from the Georgia Gulf Co. Ltd. (Atlanta, GA, USA). Titanium dioxide nanoparticles (TiO_2_-NPs), with crystalline forms of anatase (purity: 99.5%, APS: 10-30 nm, SAS: 50 m^2^/g) was supplied by Skyspring Nanomaterials (Houston, TX, USA). The polymer solvent N,N-dimethyl acetamide (DMAc) was purchased from Sigma-Aldrich Corporation (Darmstadt, Germany).

### 2.2. Membrane Preparation

To remove moisture from the PVC, it was dried in an oven at 70 °C for 4 h. Dried PVC (15 wt%) was added to 85 wt.% DMAc solvent was used to prepare the casting solution. After the casting solution became homogeneous using a magnetic stirrer for 12 h at 40 °C, the inorganic TiO_2_-NPs were added. The final casting solution, at different loads of TiO_2_-NPs (coded as PT-0 = 0, PT-0.5 = 0.5, PT-1 = 1, and PT-1.5 = 1.5 gm), was kept for 30 min in the ultrasonic to prohibit aggregation of the TiO_2_-NPs. The solution was then cast at 200 μm thickness using a motorized film applicator (CX4 mtv messtechnik, Germany) at room temperature. The glass plate was instantly immersed in pure water to complete the membrane formation. The nascent membrane was transferred to a pure water container and kept for one day to ensure that all the solvent was removed from the membrane. The membrane was kept for two days in a 30 wt.% glycerol solution to protect the membrane structure from cracking and collapsing. Three identical flat sheet membranes were selected to conduct the characterization and to examine the UF tests. The amount of TiO_2_-NPs was chosen from the literature; for example, Claudia et al. [9] found that 2 gm of TiO_2_-NPs leads to an agglomeration of nanoparticles.

### 2.3. Characterization of the Prepared Membranes

#### 2.3.1. CA Test

To quantify the CA of the PVC-TiO_2_-NPs membranes, an optical CA meter model (CAM 110-O4W, Taiwan) was used. For each membrane sample, five CA measured values were recorded, and the average CA was determined.

#### 2.3.2. Scanning Electron Microscopy (SEM) and Energy Dispersive X-ray (EDX)

A scanning electron microscope was used to investigate the properties of the cross-sectional and of the top membrane surface. With a TESCAN VEGA3 SB instrument (EO-Service, Kohoutovice, Czech Republic), SEM images were taken. The SEM instrument was equipped with an energy dispersive X-ray, which operated at higher electron voltage (eV) and provided elemental mapping to determine the elemental composition of the material. Membrane samples were prepared for the SEM test using liquid nitrogen to prevent the deformation of the cross-section of the membrane. 

#### 2.3.3. Fourier Transforms Infrared Spectroscopy (FTIR) and X-ray Diffraction

The FTIR test was conducted using an 8400S Shimadzu (Tokyo, Japan), with a wavenumber range of 400–4000 cm^−1^. X-ray diffraction was employed to obtain the phase patterns of the samples. Patterns were obtained using Cu Kα radiation, and the radiation wavelength λ = 0.1542 nm. All samples were scanned at 2θ, in the range of 10–90°, at 13 °C, with a speed of 10 (deg/min), using an XRD-6000 Shimadzu (Tokyo, Japan).

#### 2.3.4. AFM Test

The AFM device Angstrom, Scanning Probe Microscope (SPM), AA 3000 A°, Angstrom Advanced Inc., (Stoughton, MA, USA) was employed for the characterization of the surface topography 3-D image, roughness, mean pore size, distribution of pore size, and maximum pore size of the prepared membranes. PVC-TiO_2_-NPs membrane surfaces were scanned with an image size ≥ (3000 nm, 3000 nm) and topography pixels of more than 400,000.

#### 2.3.5. Tensile Test

Mechanical tests were performed using the tensile test machine (Tinius Olsen, H50 KT, Horsham, PA, USA). The load cell was 5 N at ambient temperature. Each sample had the dimensions 10 × 100 mm. A cross-head speed of 5 mm/min was used according to polymer standards.

#### 2.3.6. Porosity Test

The PVC-TiO_2_-NPs membrane porosity (ε %) was estimated by weighing the membrane sample before and after soaking it in distilled water. The water at the surface of the membrane was dried carefully before measuring the weight of the wet sample. The soaking time was 24 h at 25 °C [22]. 

The membrane porosity (ε %) was estimated by employing Equation (1).

(1) $$\varepsilon =\frac{w1-w2}{A\times T\times \rho }\times 100$$

where ε is the porosity (%), w1 is the weight of the wet sample (g), w2 is the weight of the dry sample (g), A is the effective area (cm^2^), T is the thickness of the sample (µm), and ρ is the density of water (g/cm^3^).

### 2.4. PVC-TiO_2_-NPs Membrane Performance

A Cross-flow experimental system was used to measure the permeation flux and rejection of the pollutant by PVC-TiO_2_-NPs membranes. The membrane module was purchased from Delta Company (Italy). The outer area of the membrane cell was 54.76 cm^2^, with an effective area of 18.1 cm^2^. The separation performance of the membrane can be significantly affected by the flow pattern. Therefore, a countercurrent flow pattern was used in the membrane modules. The volume of the solution was 5 L. All experiments were performed at room temperature. The PWP was estimated using Equation (2):

(2) $$\mathrm{PWP}=\frac{V}{t\times A\times P}$$

where V is the volume of the permeate (L), t is time of the collected permeate (h), A is the effective surface area (m^2^), and P is the pressure across the membrane wall (bar).

The retentate or solute rejection R (%) of the COD, TSS, turbidity, heavy metals, and oil and grease concentration was estimated by Equation (3):

(3) $$R=\left(1-\frac{\mathrm{Cp}}{\mathrm{Cf}}\right)\times 100$$

where Cf and Cp are the solute composition (mg/L) of the influent (feed solution) and permeate solution, respectively. 

**Figure 1:**
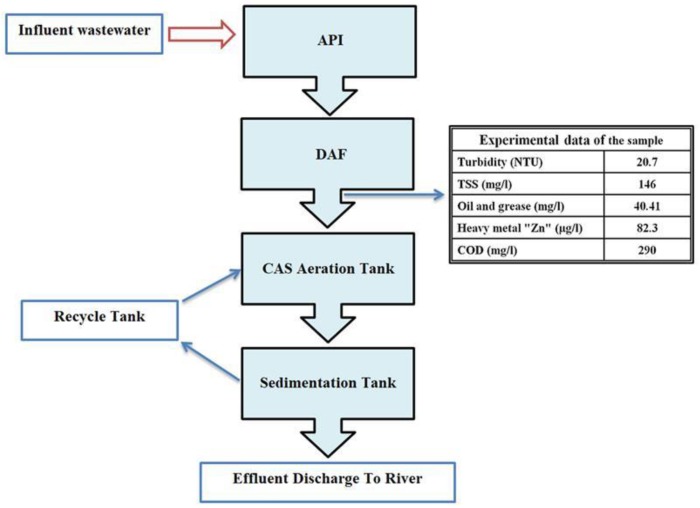
Diagram of petroleum wastewater treatment in Al-Daura refinery.

The characteristics of the influent oily wastewater (from the Al-Dura refinery, Baghdad) were COD ~290 mg/L; PH ~6.9; TSS ~146 mg/L; and turbidity ~20.7 NTU; heavy metals (Zn = 82.3 µg/L; Mn = 0); and oil and grease ~40.14 mg/L. A schematic diagram of the classical treatment processes of oily wastewater in the Al-Dura refinery is shown in Figure 1.

### 2.5. Experimental Setup

**Figure 2:**
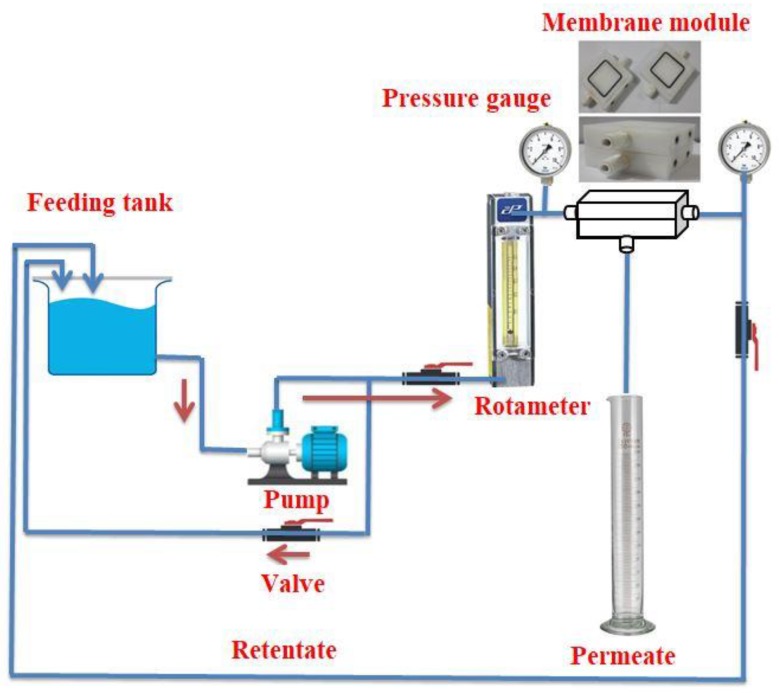
Schematic diagram of ultrafiltration membrane (UF) experimental system.

A cross-flow experimental system was utilized to perform the UF experiment for actual oily wastewater treatment, as illustrated in Figure 2. A feeding suction pump was used to pump this oily wastewater from the influent tank, with a maximum flow rate of 1.6 L/min and a maximum pressure 125 psia, operated by DC current at 24 V. This actual oily wastewater was taken from the Al-Dura refinery/Utilities Power Commission (Baghdad, Iraq) after a dissolved air flotation unit (DAF) was used to remove floating oils.

## 3. Results and Discussion

### 3.1. CA, Thickness, and Porosity

**Figure 3:**
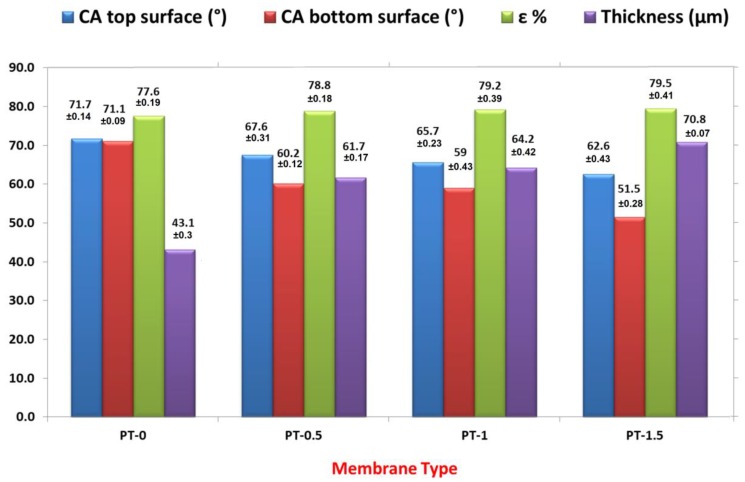
Contact angle (CA) (°), thickness (µm) and porosity (ε %) of the neat-PVC and PVC/TiO_2_NPs membranes.

The contact angles (CAs), thickness, and porosity of the neat PVC membrane and the PVC with various amounts of TiO_2_-NPs are depicted in Figure 3. On the top surface, the CA values decreased from 71.684° for neat PVC to 62.62° with TiO_2_-NP amounts of 1.5 gm, (i.e., the reduction of the CA value was about 9.06°). Therefore, it can be concluded that implanted TiO_2_-NPs have a great effect on the hydrophilic character of the PVC-TiO_2_-NPs membranes because of the hydrophilic property of TiO_2_-NPs [23]. On the other hand, the values of the CAs from the bottom surface decreased for the neat PVC from 71.091° to 51.543°, with a TiO_2_-NP amount of 1.5 gm (i.e., the reduction of the CA value was about 19.4°). According to the results of the AFM analysis, the contact angle of the bottom surface was lower in comparison with the top surface because the bottom surface was rougher than the top for all membrane types. This also resulted from the fact that NPs reduce the exchange rate between the solvent in the casting solution and the nonsolvent in the coagulation bath. In addition, the delay exchange rate was higher at the bottom surface than at the top surface, which is in direct contact with the coagulation bath [6].

The thickness of the membrane increased with the addition of TiO_2_-NPs because of the rise in viscosity of the casting solution with the rise in TiO_2_-NP levels (i.e., increasing the solid materials in the dope solution resulted in increased membrane thickness) [5].

Figure 3 displays the influence of the amount of TiO_2_-NPs on the PVC-TiO_2_-NPs membrane porosity. Increasing the TiO_2_-NPs concentration led to a moderate rise in the membrane porosity from 77.6% for the neat PVC membrane to 79.5% for the membrane prepared from TiO_2_-NPs 1.5 gm. These results were comparable with the studies presented in the literature in which the addition of hydrophilic compounds improved the porosity or void size [5].

### 3.2. EDX Analysis

Energy dispersive X-ray spectroscopy (EDX) was used to analyze the PVC-TiO_2_ elemental composition. Table 2 shows that the elements (i.e., Ti, O, Cl, and C) were detected at different weight percentages. The PVC-TiO_2_ elemental mapping displayed different elements distributed over the top surface of PVC-TiO_2_ membrane. The color mapping observed in Figure S1 (supplementary information file) confirms the presence of Ti, O, Cl, and C in the prepared PVC-TiO_2_ membrane. Scattering of nano-TiO_2_ among the entire membrane was indicated from the corresponding Ti element mappings. The most significant observation here is the optimum dispersion of the TiO_2_-NPs in the matrix of the membrane prepared from 0.5 gm TiO_2_-NPs. Also, it can be noticed that small aggregation of TiO_2_-NPs in the membrane matrix was observed with increasing TiO_2_-NPs amount in the PVC casting solution. This is attributed to the perfect dispersion of TiO_2_-NPs in PVC solution from utilizing the ultrasonic device.

### 3.3. FTIR Results

**Figure 4:**
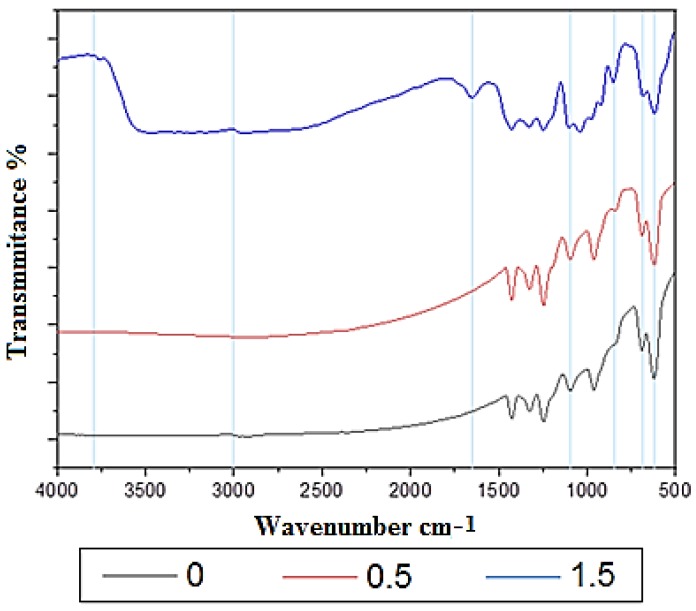
Fourier transforms infrared spectroscopy (FTIR) spectra of the PVC/TiO_2_ membranes.

The FTIR spectrum of PT-0 displayed various bands characteristic of bending vibrations and of the stretching of the O–H, C–H, C–Cl, and C=C groups. The characteristic bands of neat PVC can be divided into three regions, as seen in Figure 4. The stretching region of C–Cl is the first region, with the range 800 and 600 cm^−1^. The stretching region of C–C is the second region, with the range 1200 and 900 cm^−1^, while in the PVC, the third region ranged between 1425 and 1250 cm^−1^ [24]. As shown in Figure 4, the generation of functional groups was proved by employing FTIR spectra. From the carboxylic groups (–COOH), the peaks around 1000–780 (cm^−1^) are attributed to the stretching vibration of C=O. Moreover, the formation of carboxyl functional groups was indicated by the stretching vibration of C–H at 3000–2900 (cm^−1^). The O–H stretch of the hydroxyl group was indicated by the new peaks emerging within the 3380 and 3790 cm^−1^ range.

FTIR spectra of the modified membranes are also provided in Figure 4. The FTIR spectrum of PT-0.5 exhibited similar peaks to those found in PT-0, as shown in Figure 4, as the result of such a small concentration of TiO_2_ in PT-0.5. The absorption band located at 1650 cm^−1^ represents the stretching of titanium carboxylate. The bands between 850 and 450 cm^−1^ relate to the Ti–O stretching of the anatase structure. It can be concluded that the peaks mentioned above refer to the homogeneous grafting between the PVC and the nanofiller as well as the successful production of nanocomposite membranes. These results are comparable with previously reported results by Reddeppa et al. [24] and El Sherbiny et al. [25].

### 3.4. X-ray Diffraction Results

**Figure 5:**
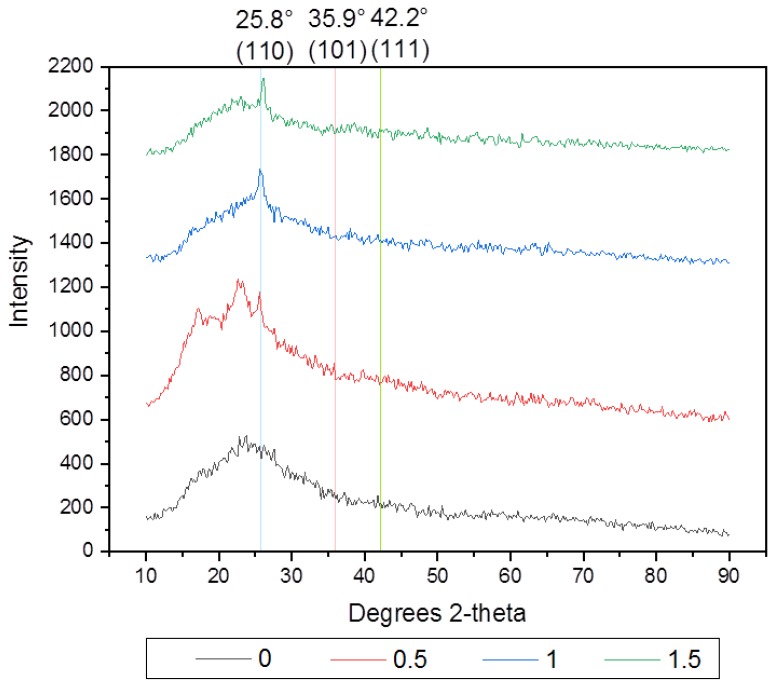
X-ray diffraction (XRD) spectra of the PVC and PVC/TiO_2_NPs membranes.

As can be seen from Figure 5, the set of the diffraction planes (110, 101, and 111) have three dominant peaks at 2θ of 25.8°, 35.9°, and 42.2° respectively, which are analogous to the crystallographic structure anatase phase of TiO_2_-NPs [8,25]. The presence of the dominant peak at 2θ of 25.8° for PT-0.5, PT-1 and PT-1.5 differs from the results for PT-0, and the values of intensity at this peak were increased with increasing of TiO_2_-NPs, where there was a broad peak corresponding to the PVC between 18.01° and 23.8°, followed by a less intense peak at 41.2°. This indicates that the TiO_2_ nanoparticles were uniformly distributed in the flat sheet membranes prepared for the phase inversion process. The peaks near 25° for TiO_2_ merged with those corresponding to the neat PVC, indicating the presence of NPs in the membranes. These results were in agreement with the results in the literature [24,25].

### 3.5. Morphology Examination by SEM

**Figure 6:**
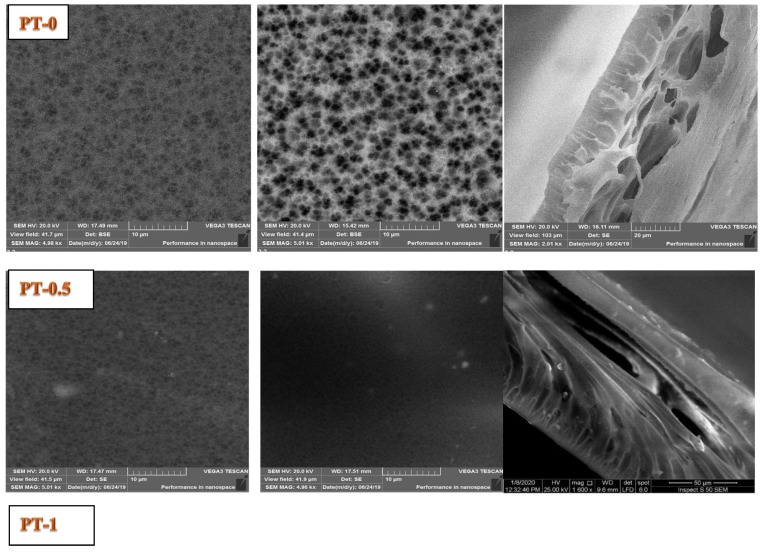

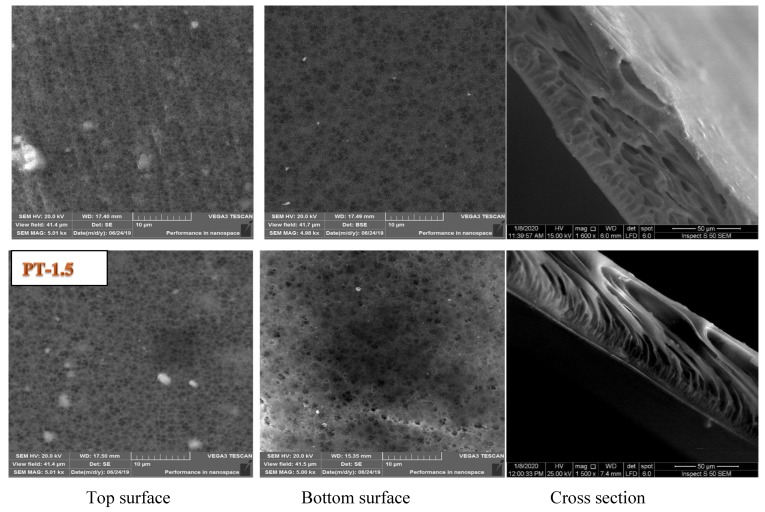
Scanning electron microscope (SEM) images of the cross-section and top and bottom surfaces of the PVC membranes at different TiO_2_ amounts: (**PT-0**) 0 gm TiO_2_; (**PT-0.5**) 0.5 gm TiO_2_; (**PT-1**) 1 gm TiO_2_; and (**PT-1.5**) 1.5 gm TiO_2_.

The morphology of the top surface, bottom surface, and the cross section of all prepared membranes were checked by SEM and are shown in Figure 6. The cross section of PT-0 has an ellipse-like structure. The addition of TiO_2_-NPs 0.5 gm to the casting solution changed the cross section from an ellipse-like structure to a finger-like structure. No significant change in the cross-sectional structure was observed with further increases in the amount of TiO_2_-NPs in the casting solution (i.e., 1 and 1.5 gm of TiO_2_-NPs). This minor change in the cross-section of the PVC membrane with addition of TiO_2_-NPs from 0.5 to 1.5 gm in PVC solution was a good indication on the optimum dispersion of TiO_2_-NPs in the matrix of the membranes. The uniform dispersion on the cross-section of the membrane and EDX observations were supports this result. It can be seen from Figure 6 that the PT-0 top surface had a high pore density and a small pore size, whereas the bottom surface was porous, and the pore size was larger than for the top surface. On the other hand, the TiO_2_-NPs produced a large effect on both surfaces of the modified membranes, as shown in Figure 6. 

The top surface of the modified membranes (i.e., PT-0.5, PT-1, and PT-1.5 of TiO_2_-NPs) had a higher pore density with a smaller pore size in comparison with the top surface of PT-0. The formation of membranes with a highly porous or dense structure depends on the exchange rate between the solvent and the nonsolvent during the phase inversion process; more porous membranes are formed when a nonsolvent enters the casting film faster than the solvent escapes [5,8,26,27,28]. The pores on the bottom surface were few and broader than the pores on the top surface but were more organized with high pore density on the top surface.

### 3.6. AFM Test

Table 3 illustrates that the mean pore size of the PT-0 top and bottom surfaces were 69 and 96 nm, respectively. The mean pore size increased to 92 and 99 nm for PT-0.5, and it reached 99 and 114 nm for PT-1, whereas the mean pore size for PT-1.5 was 77 for the top surface and 112 nm for the bottom surface. Adding TiO_2_ to the casting solution led to a change in the reciprocation rate between the solvent and nonsolvent during the formation of the membranes [5]. An increased load of TiO_2_ led to reduce the mean pore size for both surfaces of PT-1.5. This phenomenon relates to the aggregation of additional TiO_2_ on the membrane surface.

**Figure 7:**
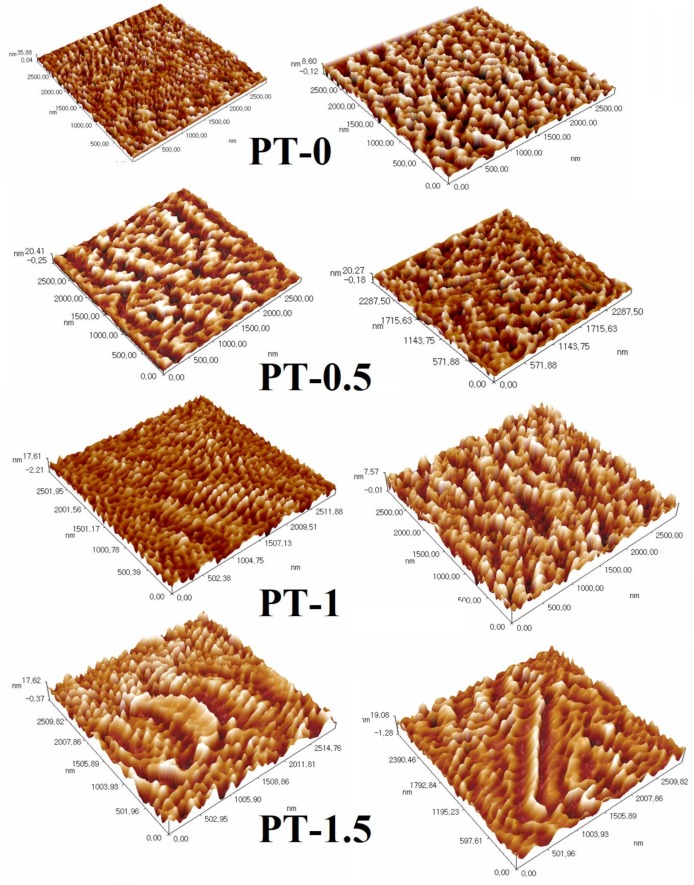
Three-dimensional (3-D) atomic force microscope (AFM) images of the top and bottom surfaces of the PVC membranes at different TiO_2_ amounts: **(PT-0**) 0 gm TiO_2_; (**PT-0.5**) 0.5 gm TiO_2_; (**PT-1**) 1 gm TiO_2_; and (**PT-1.5**) 1.5 gm TiO_2_, top surface (left) and bottom surface (right).

AFM images provided complementary information about the topography of the PVC- based membranes before and after the addition of TiO_2_-NPs, as illustrated in 3-D in Figure 7. In this 3-D image, the brightest regions represent the most elevated areas of the material surface, and the dark regions indicate the pores of the membrane. The roughness of the membrane surface is one of the key parameters that affects membrane antifouling, with better antifouling found with smoother membrane surfaces [8]. According to the AFM images and the corresponding roughness parameters presented in Table 4, the surface roughness of the modified membranes displayed an apparent decrease compared with neat PVC when in relation to the following measures: Ra (average surface roughness), Rq (root mean square), and Rz, (the space between the lowest pore and highest peak). 

The Ra value decreased from 6.48 nm (PT-0) to 2.36 nm (PT-1), the Rq value decreased from 7.9 nm (PT-0) to 2.97 nm (PT-1), and the Rz value decreased from 35.8 nm (PT-0) to 19.8 nm (PT-1), possibly due to the effect of adding TiO_2_, which makes the surface smoother. The same impact of TiO_2_ was found in the roughness of the bottom surface of the prepared membranes. Additional loading of TiO_2_-NPs resulted in a slight increase in the mean roughness to 2.82 nm for PT-1.5. The main reason for TiO_2_ makes the bottom surface rougher than the top surface was due to the delay liquid/liquid demixing process because of increasing viscosity during casting of the composite membrane. Also, this was due to the aggregation of more TiO_2_ on the membrane surface. These results support the utility of TiO_2_-NPs as a nanofiller with polymeric membranes to improve antifouling performance, which was also found by Hamid et al. [29]. 

**Figure 8:**
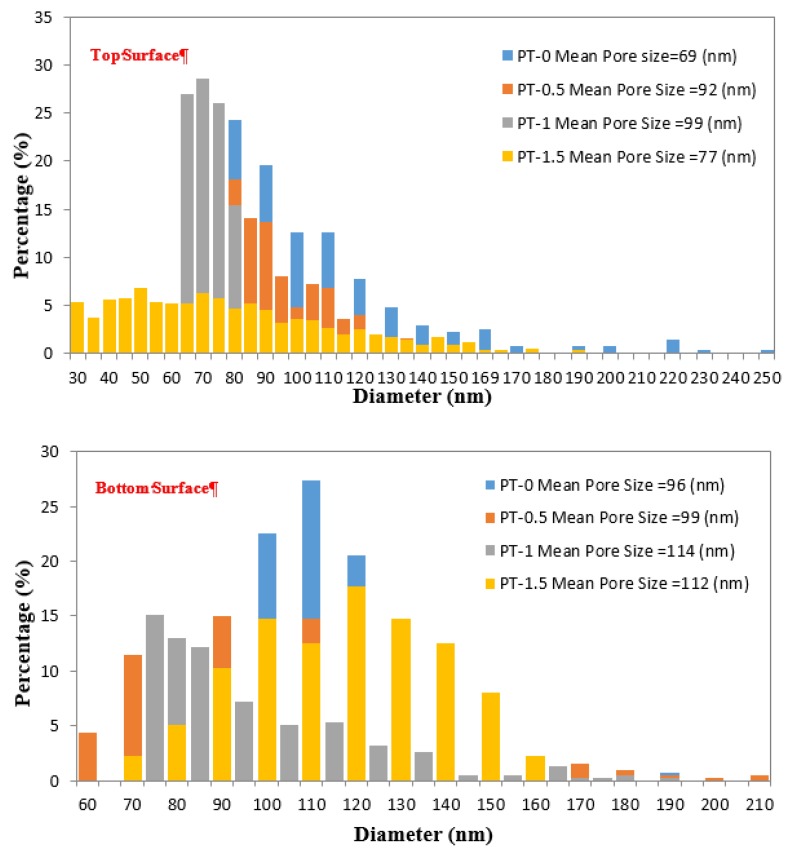
Pore size distribution of the (**top)** and (**bottom)** surfaces of the PVC membranes at different TiO_2_ amounts: (PT-0) 0 gm TiO_2_; (PT-0.5) 0.5 gm TiO_2_; (PT-1) 1 gm TiO_2_; and (PT-1.5) 1.5 gm TiO_2_.

The cumulative distribution percentage of the pore sizes on the surface of the PVC-TiO_2_-NPs increased with narrow distribution in comparison to that of the neat PVC membrane, as can be seen in Figure 8. High pore sizes with a narrow distribution of the pore size were obtained with an increase of TiO_2_-NPs, up to 1.5 gm. Membranes with a highly porous structure are formed when a nonsolvent enters the casting film faster than the solvent escapes, as was mentioned in Section 3.5 (“Morphology examination by SEM”).

### 3.7. Mechanical Tensile Test

Compared to many other polymers, PVC has excellent mechanical properties, giving the PVC membrane the strength to deal with transmembrane pressures. The tensile strength at break of the PVC-TiO_2_-NPs membranes was higher than 2 MPa, as shown in Table 5. Implanting TiO_2_-NPs in the membranes led to a slight improvement in the tensile strength of the PVC-TiO_2_-NPs membranes because of the reinforcement effect of TiO_2_-NPs. The PT-0 tensile strength was 2.098 MPa, which increased to 2.281 MPa for PT-1.5. This may have been due to the uniform dispersion of the TiO_2_ nanoparticles in the polymer solution during the synthesis of membranes, which leads to homogeneity of the casting solution and increased tensile strength. These results closely resemble the results of Yu et al. [10] and Alsalhy et al. [5]. It is worth to mention here that the improvement in tensile strength makes the composite PVC-TiO_2_-NPs membranes less prone to tearing during handling.

### 3.8. Performance of the Membranes (Short-Term and Long-Term)

**Figure 9:**
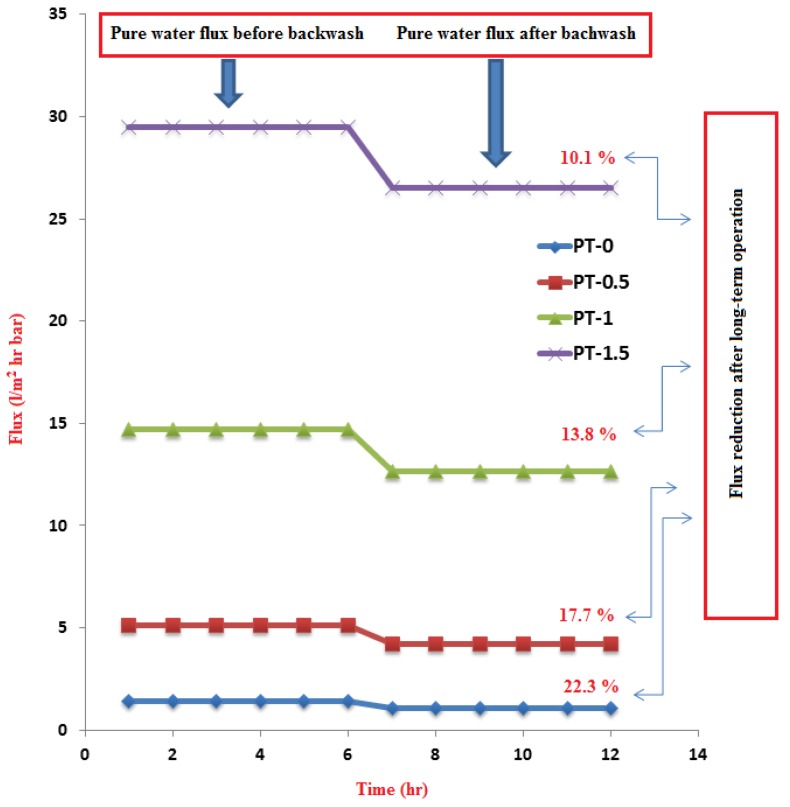
Flux of pure water before and after backwashing for 12 h at pressure of 2 bar and percentage of flux reduction of the PVC membranes at different TiO_2_ amounts: (PT-0) 0 gm TiO_2_; (PT-0.5) 0.5 gm TiO_2_; (PT-1) 1 gm TiO_2_; and (PT-1.5) 1.5 gm TiO_2_.

The effects of various TiO_2_-NPs loadings on the PWP of the PVC-TiO_2_-NPs membranes before and after backwashing at room temperature during 6 h of operation are shown in Figure 9. The PWP of the neat PVC membrane was 1.392 L/m^2^·h, whereas implanting TiO_2_-NPs in the PVC solution enhanced the PWP to 5.113 L/m^2^·h for the membrane with PT-0.5, 14.732 L/m^2^·h for PT-1, and 29.511 L/m^2^·h for PT-1.5. This finding can be attributed to the improvement in the hydrophilic character of the composite membrane as a result of adding various amounts of TiO_2_-NPs.

**Figure 10:**
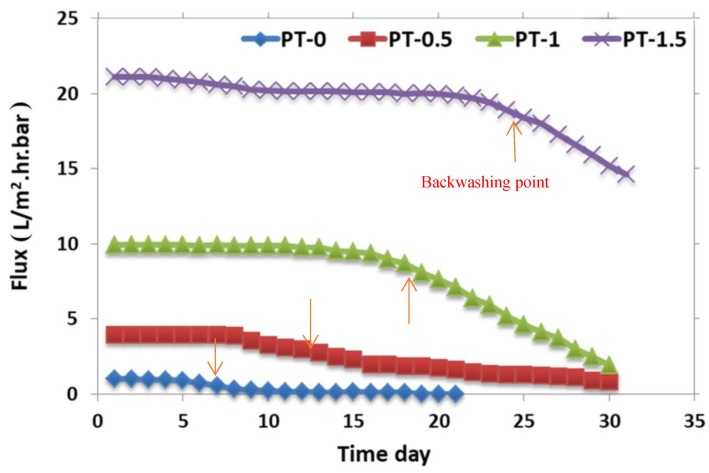
The effect of TiO_2_NPs in casting solution on long-term of the prepared membranes: (PT-0) 0 gm TiO_2_; (PT-0.5) 0.5 gm TiO_2_; (PT-1) 1 gm TiO_2_; and (PT-1.5) 1.5 gm TiO_2_, by using oily wastewater with characteristics of chemical oxygen demand (COD) ~290 mg/L; PH ~6.9; total suspended solid (TSS) ~146 mg/L; and turbidity ~20.7 NTU; heavy metals (Zn = 82.3 µg/L; Mn = 0); and oil and grease ~40.14 mg/L.

The membrane’s ability to endure long term was evaluated by passing oily wastewater through the membrane, as shown in Figure 10. It can be seen that PT-0 started to decay after 5 d, and the membrane worked for more than 14 d total before membrane cleaning was required. The permeation flux of PT-0.5 started to decrease after 10 days, and this membrane worked for more than 30 d before cleaning was required. The permeation flux of PT-1 started to decrease after 17 d, and this membrane worked for more than 30 d before cleaning was required. The last membrane, PT-1.5, started to decay after 23 d but worked for more than 30 d before cleaning was required (see Figure 10). These outcomes clearly demonstrate that fouling was inversely proportional to the TiO_2_-NPs amounts.

After completing the experiment on the long-term membrane performance, the flat sheet membranes were backwashed using pure water for 6 h at 2 bar, and pure water fluxes were again measured. As shown in Figure 9, the pure water flux after backwashing was lower than before backwashing; this is due to the retention of contaminants on the surface of each membrane because some of the contaminants being held in the membrane cannot be completely removed by backwashing. In Figure 9, the PWP reduction of the neat PVC membrane (P-0) after backwashing was 22.3%, while the PWP was reduced to 17.7% by the addition of TiO_2_-NPs 0.5 gm. A further increase in TiO_2_-NPs to 1 and 1.5 gm reduced the PWP by 13.8% and 10.1%, respectively. These results clearly identify the impact of the TiO_2_-NPs content on the long-term PVC-TiO_2_ membrane performance and also confirm our hypothesis that it is possible to employ TiO_2_-NPs to effectively enhance the lifetime of the membranes during their long-term operation and overcome the significant effect of membrane fouling. The mitigation of the membrane fouling was largely affected by TiO_2_-NPs surface area, which were existed on the surface of the membrane and directly exposed to inlet feed of wastewater and the level of fouling mitigation was mainly depended on the concentration of TiO_2_-NPs at the surface of the membrane. Therefore, the improvement of the PVC-TiO_2_-NPs membranes performance may be caused from this phenomenon. By implanting nanoparticles for wastewater treatment, He and Jiang [30] found similar effects in the membrane structure.

**Figure 11:**
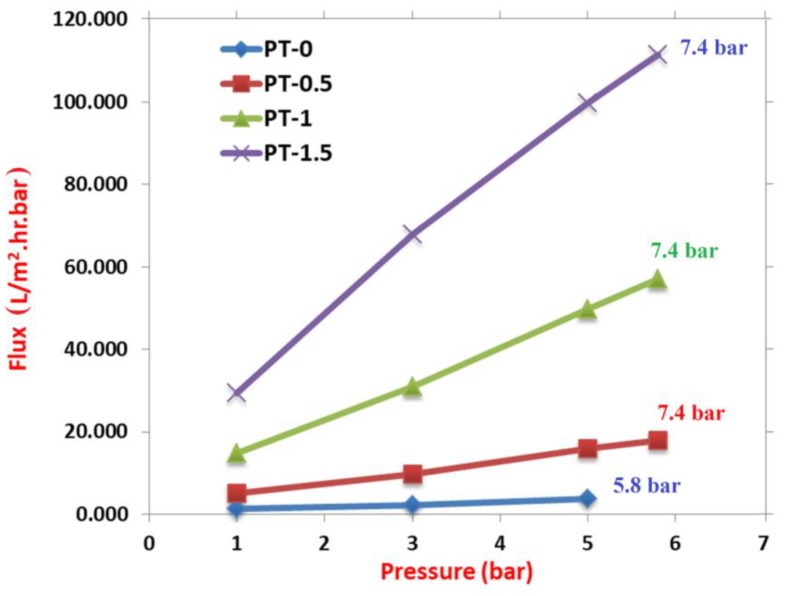
Effect of the feeding pressure on the pure water flux of the PVC membranes at different TiO_2_ amounts: (PT-0) 0 gm TiO_2_; (PT-0.5) 0.5 gm TiO_2_; (PT-1) 1 gm TiO_2_; and (PT-1.5) 1.5 gm TiO_2_.

The membrane pressure tolerance was investigated using pure water as a feed to the UF system at room temperature with variations in the transmembrane pressure from 1 to 7.4 bar. As shown in Figure 11, the pure water flux for PT-0 increased from 1.392 to 2.227 L/m^2^·h with an increase in the feeding pressure to 3 bar and rose to 3.758 L/m^2^·h at 5 bar. However, implanting TiO_2_-NPs in the membrane body increased the pure water flux threefold, to 5 bar, in comparison with the 1 bar increase for PT-0.5, PT-1, and PT-1.5, where the pure water fluxes reached the values of 15.876, 47.408, and 94.701 L/m^2^·h, respectively. As presented in Figure 11, the modified membranes with the highest pressure tolerance reached 7.4 bar in comparison with the unmodified membrane PT-0, where the pressure tolerance test failed at 5.8 bar. This result shows that the pressure tolerance is directly affected by the reinforcement impact of TiO_2_-NPs, which enhance the transverse strength of the membrane structure. This phenomenon agreed with the results of the tensile strength test and was also found by Yu et al. [10].

**Figure 12:**
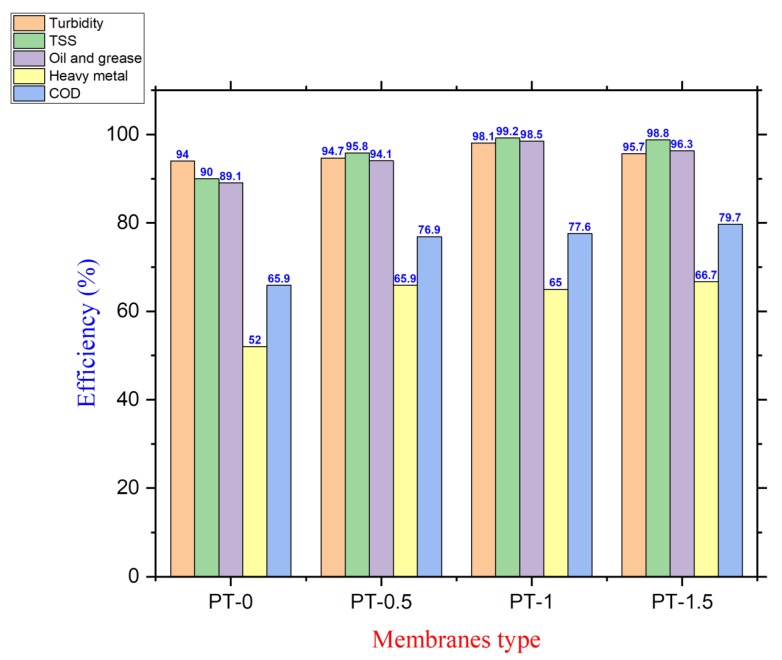
Turbidity, TSS, oil and grease, heavy metal and COD removal efficiency of the PVC membranes at different TiO_2_ amounts: (PT-0) 0 gm TiO_2_; (PT-0.5) 0.5 gm TiO_2_; (PT-1) 1 gm TiO_2_; and (PT-1.5) 1.5 gm TiO_2_, by using oily wastewater with characteristics of COD ~290 mg/L; PH ~6.9; TSS ~146 mg/L; and turbidity ~20.7 NTU; heavy metals (Zn = 82.3 µg/L; Mn = 0); and oil and grease ~40.14 mg/L.

Figure 12 shows the turbidity, TSS, oil and grease, heavy metals, and COD removal efficiency of the prepared membranes at room temperature as well as the flow rate (1.1 L/min) and pressure (1 bar) during 48 h of operation. The turbidity removal efficiency increased with increasing amounts of TiO_2_-NPs in the membrane body, and PT-1 had the highest turbidity removal efficiency (98.1%). Implanting TiO_2_-NPs in the PVC solution modified the structural morphology of the PVC-TiO_2_-NPs membranes. For example, the addition of nanoparticles significantly decreased the roughness of the membrane surface. Also, the high pore density on the surface of the membranes provided more vacant sites that attracted a huge amount of contaminants, including dispersed and suspended solids, clay, organic matter, silt, algae, and other microorganisms in a short time, which decreased the cloudiness of the solution after the filtration process [31]. The UF membrane is capable of handling high levels of suspended solids with excellent removal efficiency because most of the suspended solid materials have a size larger than the pore diameter of the membrane [31,32]. 

Moreover, from Figure 12, the TSS removal efficiency improved with the implantation of TiO_2_-NPs in the PVC solution. TSS removal efficiency increased from 90% for neat PVC to 99.2% and 98.8% for PT-1 and PT-1.5, respectively. As can be seen in Figure 12, oil and grease rejection were improved from 89.1% for PT-0 to 98.5 and 96.3% for PT-1 and PT-1.5, respectively. Oil and grease particulates were completely rejected when the content of the oil found in the permeate was less than 3 mg/L. This oil content was within the allowable discharge concentration (4 mg/L) [7,31,32]. 

In addition, the heavy metal removal efficiency represented by Zn(II) rejection for the neat PVC and PVC-TiO_2_-NPs membranes is also displayed in Figure 12. Generally, a membrane works as a sieve to remove molecules according to their size. Only particles that are larger than the pore size are retained. In this case, heavy metals, represented by Zn(II), were removed with the following efficiency levels for PT-0, PT-0.5, PT-1, and PT-1.5: 52%, 65.9%, 65%, and 66.7%, respectively. The Zn(II) removal efficiency of neat PVC membranes was lower in comparison with the PVC-TiO_2_ membranes, which was clearly due to increasing the modified membrane’s thickness, thus providing a larger surface area. These are regarded as good adsorbents for the removal of heavy metal ions and other inorganic substances, with similar results found by Khulbe and Matsuura [33].

As shown in Figure 12, the membrane PT-1.5 exhibited the highest COD rejection with 79.66%. It was observed that the removal efficiency of COD was approximately 65.86% for the neat PVC membrane and more than 75% for all membranes prepared with various TiO_2_-NPs amounts. The ultrafiltration system worked to remove the suspended solids with high efficiency as confirmed by the results of the TSS tests. And the organic matter was either dispersed in the wastewater in a soluble form, in colloidal minutes, or it adhered to the outer surface of the suspended solids. The removal of the suspended solids will lead to partially removing the organic matter, which also explains the improvement in the COD rejection. On the other hand, the ultrafiltration of the semipermeable membrane with a pore size of 0.005–0.1 µm retained some organic material that was larger than the pore size of the membrane, which reduced the amount of organic material in the permeate. The improvement in the membrane hydrophilicity by adding TiO_2_-NPs was another reason for the enhancement in the COD rejection [5,32].

Finally, according to the Iraqi Law No. 25 in 2010 were listed in Table 6, it is worth to mention here that acceptable limits of all of the contaminants in the discharged water to the river [34].

Table 7 shows a comparison between the PVC-TiO_2_-NPs membranes performance fabricated by the present work with the performance of selected values of prepared and commercial membranes found from the literature. The most important characteristics of the membranes such as mean pore size, porosity, and contact angle were also presented in Table 7. It can be noticed that the PVC-TiO_2_-NPs membranes has a reasonable PWP and pollutants removal efficiency as well as high flux recovery ratio (%) in comparison with most membranes found in the literature.

## 4. Conclusions

Due to the excellent properties of PVC polymer and TiO_2_-NPs, the TiO_2_ was successfully implanted in the PVC membranes. With increasing amounts of TiO_2_-NPs, the CA of the membrane (indicating its hydrophilic property) improved by 12.7% and 27.5% on the top and bottom faces of the membrane, respectively. This is attributed to the increasing of TiO_2_-NPs amount towards the surface during membranes formation, which in turn led to enhance the hydrophilicity of the surface. Moderate changes in structural morphology and porosity were observed due to the impact of TiO_2_-NPs on the viscosity of PVC solution and the liquid-liquid mass exchange during the membrane formation. The pure water permeability (PWP) of the PVC-TiO_2_-NPs membrane was enhanced by 95.3% with the addition of TiO_2_-NPs. With the addition of TiO_2_-NPs, the PWP after backwashing decreased from 22.3% for neat PVC to 10.1%. The long-term performance was improved from 5 days for neat PVC to 23 d with increasing amounts of TiO_2_-NPs due to the improvement in tensile strength, which makes the composite PVC-TiO_2_-NPs membranes less prone to tearing during handling. From the results of the current work, it can be concluded that the TiO_2_-NPs content significantly impacted the long-term PVC-TiO_2_-NPs membrane performance and greatly extended the lifetime of the membranes during their long-term operation. Also, incorporation of TiO_2_-NPs led to improve the fouling resistance of PVC-TiO_2_ membranes and to enhance the performance of the composite membranes in relation to the turbidity, TSS, oil and grease, heavy metals, and COD rejection.

## Figures and Tables

**Table 1:** Studies have reported the embedded of TiO_2_-NPs with several polymers and cellulose acetate.

Polymers	Process	Nanoparticles		Reference
		Name	Content	
PVC	UF	(Ag-n-TiO_2_)	0 to 1.5 wt%	[10]
PVDF	MF	TiO_2_	0.05 wt. %	[13]
PVDF	UF	TiO_2_	0.1 g/l	[14]
PVDF	UF	TiO_2_	0.5–1 wt. %	[15]
PVDF	UF	TiO_2_	0–6 wt %	[16]
PP	UF	TiO_2_	---	[17]
PSF	UF	TiO_2_	0.1, 0.25 and 0.5 wt. %	[18]
CAC	UF	TiO_2_	0–25 wt.%	[11]
PES	NF	TiO_2_	0.125 gm	[19]

**Table 2:** Energy dispersive X-ray (EDX) analysis of wt.% of the elements in PVC-TiO_2_ membranes.

Element	TiO_2_ Content		
	0.5 (mg)	1 (mg)	1.5 (mg)
C	64.35	56.92	36.36
Cl	27.65	32.36	43.64
O	6.89	9.17	7.89
Ti	1.11	1.54	12.11

**Table 3:** Effect of TiO_2_ concentration on the mean pore size of the top and bottom surfaces of the prepared membranes.

Membrane Type	Mean Pore Size (nm)	
	Top Surface	Bottom Surface
PT-0	69	96
PT-0.5	92	99
PT-1	99	114
PT-1.5	77	112

**Table 4:** The surface roughness parameters from AFM image.

Materials Type	Rz (nm)		Rq (nm)		Rz (nm)	
	Top	Bottom	Top	Bottom	Top	Bottom
PT-0	6.48	2.18	7.9	2.52	35.8	8.62
PT-0.5	4.6	3.8	5.42	4.6	20.7	20.5
PT-1	2.36	1.49	2.97	1.78	19.8	7.58
PT-1.5	2.82	2.74	3.46	3.46	18	17.7

**Table 5:** Tensile strength of prepared membranes.

Materials Type	Tensile Strength
PT-0	2.098 ± 0.21
PT-0.5	2.254 ± 0.43
PT-1	2.269 ± 0.37
PT-1.5	2.281 ± 0.51

**Table 6:** Specifications of influent, effluent water of PVC-TiO2NPs membranes and the allowable limits of Iraq for river discharged water.

	Influent	PT-0	PT-0.5	PT-1_Effluent	PT-1.5	Allowable Limits
Turbidity (NTU)	20.7	1.25	1.1	0.4	0.9	5
TSS (mg/L)	146	14.6	6.2	1.1	1.8	60
Oil and grease (mg/L)	40.41	4.4	2.4	1	1.5	10
Heavy metal “Zn” (mg/L)	82.3	0.039	0.028	0.028	0.027	2.0
COD (mg/L)	290	99	67	65	59	100

**Table 7:** Comparison between the performances of membranes prepared in this study with various membranes found in the literature in terms of total pure water flux, removal efficiency, and flux recovery after long-term operation.

Membrane Material	Type and Amount of Additive	Mean Pore Size (nm)	Porosity (%)	Contact Angle	Flux Recovery Ratio (%)	Removal Efficiency (%)	PWP (L/m^2^·h)	Ref.
PVC (15 wt.%) (Flat sheet)	MWCNT-g-GO (0.119 wt.%)	259	81.4	13.9°	-	COD: 88.9	254	[3]
PVDF (16wt.%) (Flat sheet)	TiO_2_ (˂ 2 wt.%)	47.3		76°	-	* BSA:100	111.7	[8]
PSF/PVP; 18:5 wt/wt% (hollow fiber)	TiO_2_ (2 wt.%)	53.82	71.7	_	-	* (HA) ˃90%	120.11	[29]
NF-1 NE2540-70 NF-2.NE2540-90 (SAEHAN Corp., Korea) Polyamide (supported by PSf+polyester)		0.29 0.18			Long-term operation of 8 h With Stable final flux	Oily wastewater COD: 69 COD: 84	32 29	[35]
PVDF; 19 wt.% (hollow fiber)	TiO_2_ (1.95 wt.%)	34.05	85.41	50	81.7	Synthetic refinery wastewater: 98.83	82.5	[36]
* EPVC/PEG; 15:4 wt./wt.%	TiO_2_ (2 wt.%)	25	78.7	57.2°	81	BSA: 98	435	[37]
PVC (15 wt.%) (Flat-sheet)	TiO_2_: 1.5 gm	77	79.5	62.5°	89.9	Oil and grease: 96.3 COD: 79.7 TSS: 98.8	116	This work

* BSA: Bovine serum albumin; HA: Humic acid; EPVC: Emulsion poly(vinyl chloride); PEG: polyethylene glycol.

## Supplementary Materials

The following are available online at https://www.mdpi.com/2077-0375/10/4/77/s1, Figure S1: title, EDX color mapping of elemental analysis of the PVC/TiO_2_ membrane.
