# Supplementary material for: Experimental Investigation of the Effect of Implanting TiO2-NPs on PVC for Long-Term UF Membrane Performance to Treat Refinery Wastewater
Source: Membranes (Basel). 2020 Apr 21;10(4):77. doi: 10.3390/membranes10040077 (PMC7231373; doi:10.3390/membranes10040077)
Supplement: Supplementary file 1 [file membranes-10-00077-s001.pdf]

Supplementary information document

Experimental investigation of the effect of implanting TiO_2-NPs on PVC for long-term UF membrane performance to treat refinery wastewater

Faris H. Al-Ani[1], Qusay F. Alsalhy[2] and Rawia Subhi Raheem[1], Khalid T. Rashid[2], Alberto Figoli[3]

[1] Civil Engineering Department, University of Technology, Alsinaa Street 52, Baghdad, Iraq
[2] Membrane Technology Research Unit, Chemical Engineering Department, University of Technology, Alsinaa Street 52, Baghdad, Iraq
[3] Institute on Membrane Technology, National Research Council (ITM-CNR), 87030 Rende (CS), Italy
Corresponding authors: Prof. Dr. Qusay F. Alsalhy; E-mail: qusay_alsalhy@yahoo.com; 80006@uotechnology.edu.iq, Mobile phone: +964-7901730181

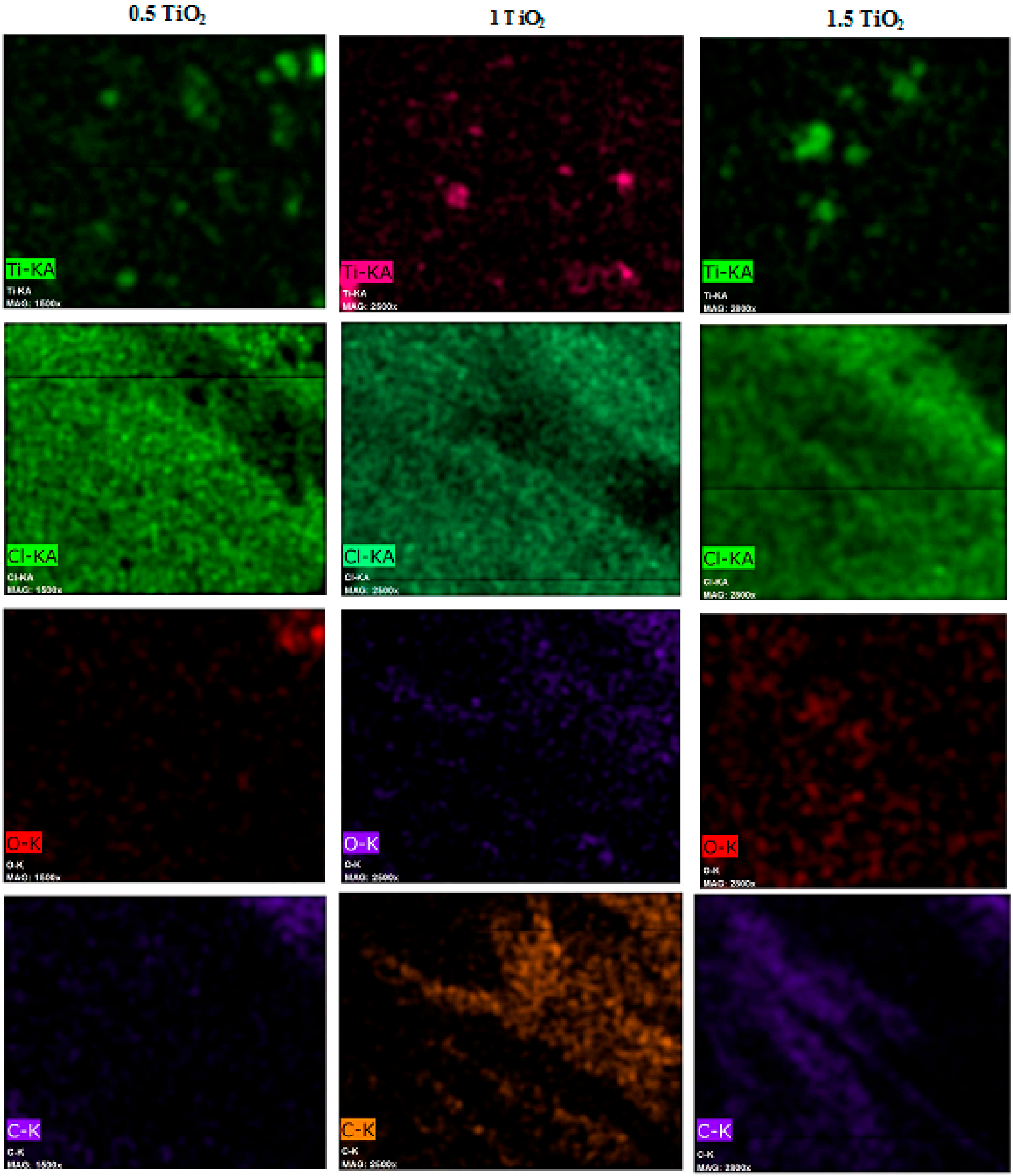

Figure S1. EDX color mapping of elemental analysis of the PVC/TiO2 membrane.
